# Callus formation during healing is guided by local strain: a retrospective clinical observation

**DOI:** 10.1186/s12891-026-10118-2

**Published:** 2026-06-26

**Authors:** Mark Heyland, Dominik Deppe, Matteo Gabriele, Julian N. Zierke, Marko Leskovar, Marie-Jacqueline Reisener, Yuriy Rozhko, Katharina Ziegeler, Philipp Damm, Simon Reinke, Ulrich Stöckle, Georg N. Duda, Stefan Zachow, Adam Trepczynski

**Affiliations:** 1 Julius Wolff Institute, Berlin Institute of Health at Charité - Universitätsmedizin Berlin, Berlin, Germany; 2 Department of Radiology, Charité – Universitätsmedizin Berlin, Berlin, Germany; 3 Zuse Institute Berlin, Berlin, Germany; 4 Center for Musculoskeletal Surgery (CMSC), Charité - Universitätsmedizin Berlin, Berlin, Germany

**Keywords:** Fracture healing and fracture fixation, In vivo loading, Interfragmentary shear strain, Callus, Femur and tibia, Intramedullary nail and locking plate, Finite element modelling, Mechanobiology

## Abstract

**Background:**

Clinically, fracture healing is typically monitored though serial radiographs. Specifically, callus development (growth and mineralization) is an indicator of healing and associated with local mechanical strain. However, a sustainable relationship between mechanical conditions and the respective healing progress has not been shown so far.

**Material and methods:**

One hundred sixty-six patients with extra-articular lower-limb fractures treated by osteosynthesis plates or intramedullary nails were included. Callus formation (visible area in X-ray relative to bone shaft) and quality (image intensity relative to the cortex) were measured by consecutive X-ray analyses as well as the modified Radiographic Union-Score for Tibia (mRUST) during follow-up. Corresponding load- and fixation-matched finite element analysis (FEA) modelling was developed for tibia or femur loading as well as plate or intramedullary nail fixation. Mechanical strains (medially, laterally, dorsally, anteriorly) were evaluated from FEA and compared to the progress in X-ray callus formation and quality to perform a correlation analysis between observed callus formation and simulated local mechanical strains.

**Results:**

For femoral fractures, callus size was largest dorso-medially (1.41 ± 1.57 cm^2^/cm and 1.18 ± 1.11 cm^2^/cm at 180 ± 45 days post-surgery) while largest callus formations were found laterally in tibial fractures (0.75 ± 0.49 cm^2^/cm at 365 ± 45 days post-surgery). These locations of maximal callus size in femur and tibia matched the locations of extreme principal strains from FEA. In femur, callus density increased steadily and exceeded cortex density at 365 ± 45 days post-surgery. For tibia, no such clear trend was observable. While initially showing a similar increase in callus bridging score mRUST, increase over 2 years was 48% higher for the tibial fractures compared to femoral fractures. While principal strains correspond to increases in early callus formation in both femur and tibia (Kendall-Tau-b: *p* = 0.021 for volumetric strain at 90 ± 45 days post-surgery), shear strains are consistently associated with less callus formation (Kendall-Tau-b: *p* = 0.048 for volumetric/shear strain associated with callus size*density at 365 ± 45 days post-surgery).

**Conclusions:**

Callus formation during bone healing may be associated with local mechanical strain in lower limb fractures within a clinically relevant cohort including different fracture locations and fixation types. Shear strain at the fracture site appeared to be associated with reduced quality callus formation, whereas principal strain was observed to correlate with increased early callus formation. The presented methodology may have potential as a predictor of healing processes and could help identify mechanically challenging fracture fixation settings.

**Level of evidence:**

II.

**Trial registration:**

Ethical approval was obtained from the local ethics board to this retrospective study design (EA4/099/24).

**Supplementary Information:**

The online version contains supplementary material available at 10.1186/s12891-026-10118-2.

## Introduction

Various technologies to stabilize a lower limb fracture have been developed that allow treatment of long bone fractures, such as compression plating, bridge plating, or intramedullary nailing. Despite the advancement and innovation in technologies, the rate of delayed bone healing and non-unions remains high with up to 10–15% of all fracture cases [1]. Beside biological factors [2, 3], a persistent challenge in the management of these fractures lies in achieving local mechanical stability at a specific fracture site by a chosen fixation technique and in understanding its subsequent impact on the progressing of fracture healing. Depending on the fracture’s localization and configuration, there are two different types of bone healing. In primary bone healing, tiny gaps (< < 1 mm) between rigidly fixed fracture fragments can be directly bridged. In secondary bone healing, fractures stabilized with relative motion between fragments heal through callus formation. Various parameters have been discussed to determine the local strains at fracture sites (Table 1),and the interaction of those parameters yields interfragmentary constraints that lead to relative motion at the fracture site, which crucially affects bone healing progress [2, 4–15].

**Table 1:** Sensitive, interacting mechanical parameters that affect the local loading and the strain (as the independent predictor of healing) at the fracture

Parameter	Details	Examples
Type of fixation and its configuration	Position, type, and design	intramedullary nail or plate or external fixator
	Compression or bridging	compression, neutralization, or locked bridging plate
	Implant material	steel or titanium
	Placement	medially, antero-lateral or lateral plate placement
Type of the fracture and reduction quality	Remaining inter-fragmental gap size	small even gap, uneven large gap, segmental defect
	Bony support under load	medial or lateral contact
	Degree of comminution	number and size of fragments
	Localization within the body	tibia or femur
	Fracture position within the bone	proximal, mid-shaft, distal
Patient’s activity and coordination level	Type of activity	walking, chair rise, stair descent, stumbling
	Muscle activation/coordination	muscle damage, dementia, neuropathies
	Use of walking aids	walker, crutches and the ability to use it properly
	Weight/BMI	obesity
	Gait parameters	speed, symmetry, step width
	Leg alignment	hip-knee-alignment angle, varus/valgus

While the individual patient aspects such as activity level or smoking may not be directly addressable by the surgeon, the mechanical conditions under which fracture healing will occur can be controlled intra-operatively by selecting an appropriate, adapted fracture fixation [16–21]. This preoperative choice of treatment has a huge impact on the healing progress by affecting the visible callus size and density (image intensity) in X-ray images but also the (invisible) maturing tissue [22–25].

Next to the implant placement and selection of screw placements, also the implant design, dimensions and material impacts the local mechanical conditions at the fracture site. Specifically, increased strain values due to flexible titanium plates can enhance callus size and formation compared to stainless steel plates [19, 22]. Such increase of callus formation is seen as being essential for bridging and stabilizing a fracture and is indicative for successful healing. Impaired callus formation is associated with a healing delay or failure such as non-union [22, 26].

Beside physical examination, X-ray imaging enables monitoring of healing progress by accessing callus formation and visibility of a fracture line in follow-up. However, there is a large variability in the appearance of callus tissue depending on the localization and the type of fracture (e.g. anterior/posterior, medial/lateral of a long bone fracture), the size and density of callus tissue, and their changes during the healing process. Using fracture healing scoring systems, like the modified Radiographic Union Score for Tibia fractures (mRUST), the healing process can be monitored via a sequence of consecutive X-ray images [27–29]. However, mRUST or similar scoring systems do not take any differences in callus formation and quality or the mechanical conditions at a fracture site into account. This subjectivity and lack of objective biomechanical data are well-documented problems in fracture assessment, driving the need for more quantitative evaluation methods, such as the in vivo load-share measurements proposed by Fu et al. [30] or the strain-correlation analysis in the present study.

In surgical fracture treatment, delayed healing is frequently explained by mechanical reasons. In pre-clinical and in silico analyses, the lack of healing has been causally related to local tissue straining or tissue deformations [6, 10, 31–35]. There is a large number of very specific assessments, mostly in animals like mice, rats, and sheep, that mechanically characterize bone regeneration processes [6, 31, 32, 34–37]. Indeed, a recent scoping review by Rechter et al. [38] confirms that while small-to-moderate axial motion appears beneficial, the definitive range of mechanical stimuli conducive to optimal healing in clinical practice remains elusive, with significant heterogeneity in the literature precluding a clear consensus. It has not been shown for a diverse, representative clinical cohort, that patient-specific callus formation during fracture healing is related to mechanical parameters. Specifically, not in a retrospective manner and in a diverse, representative cohort of fractures at different localizations, with different fixation types, and varying patient histories. If such a relationship were identified, it would not only substantiate the pre-clinical observations but also illustrate the capability to use such analyses as a means to identify mechanical ranges for success (or failure) of healing.

Aim of the present study was to evaluate callus formation and quality of distinct, but diverse lower-limb fracture situations within subsequent X-ray images with a matching biomechanical load estimated in these fracture settings to identify a correlation between biomechanical constraints at a given fracture setting and the corresponding radiographic documented callus formation progress or lack thereof. The scope of this study was to transfer and confirm the general relationships of strain and callus formation found in animal models to real human clinical cases.

## Material and methods

### Patients

**Fig. 1:**
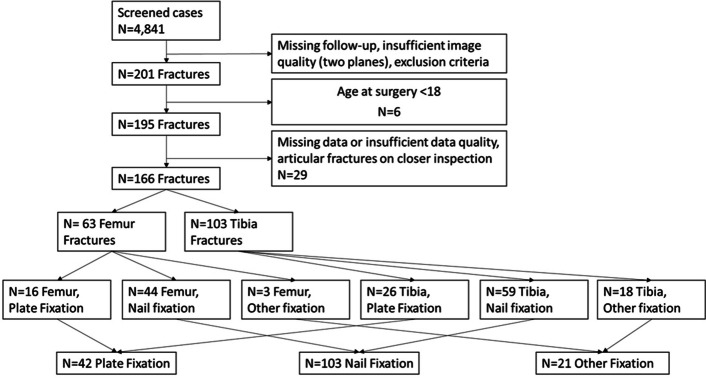
Flow diagram of the patient inclusion and exclusion due to different reasons, and resulting sub-group sizes. Overall, 166/4,841 = 3.4% of screened retrospective cases yielded sufficient data quality for this study

Retrospectively, images from the database of the Department of Radiology at Charité – Universitätsmedizin Berlin, Germany for extra-articular fractures of the lower-limb (femur, tibia) between January 2005 and April 2022 were collected. Ethical approval was obtained from the Ethics Committee of Charité – Universitätsmedizin Berlin, Berlin, Germany (EA4/099/24). We included patients > 18 years of age, with adequate X-Ray assessment (two perpendicular planes; at least one follow-up) of extra-articular fractures of the tibia or the femur. Exclusion criteria were critical clinical conditions at the time point of surgery, pregnancy or lactating women, patients who were not legally competent, intra-articular fractures (including proximal femur/femoral neck) and inadequate imaging or lack of follow-up (Fig. 1).

### X-ray assessment

**Fig. 2:**
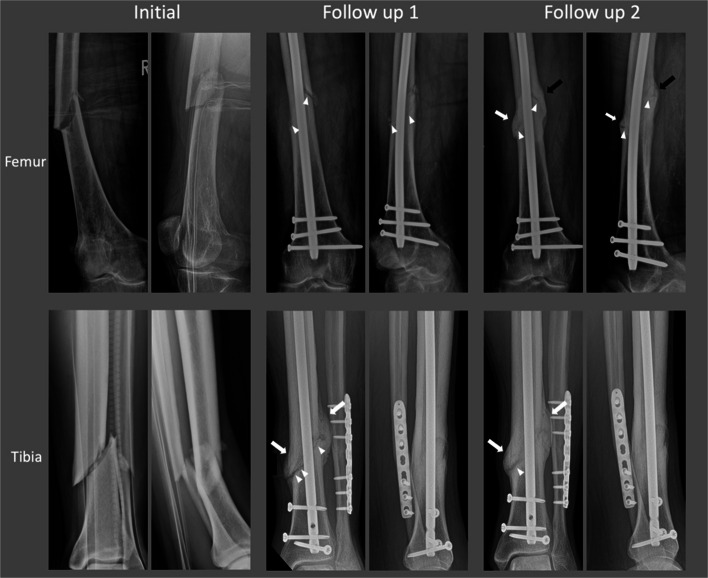
Examples of femur (top) and tibia (bottom) long bone fractures in X-rays with their corresponding follow-up images during healing (from left to right). Top: Femoral shaft fracture treated using nail fixation. No callus is seen at first follow-up image and the fracture line is still visible (white arrowhead). At the second follow-up, a callus bridging is seen at all cortices, however the dorsal and medial callus (black arrow) appears larger than lateral and ventral (white arrows). Bottom: tibial shaft fracture and fracture fixation with nailing and fibular plate. Callus (white arrows) and fracture line (white arrowhead) is seen in the first follow-up. At the second follow-up, the callus appears denser compared to its follow-up and the fracture line remains visible

Callus formation, callus quality, and fracture healing progress were assessed in X-ray images (Fig. 2) by a resident radiologist with 3 years of experience in musculoskeletal imaging. The radiological reader was blind to clinical data, timepoints and outcome. However, blinding for sex was not given in all patients due to the displayed anatomical region. Only univocal callus without overlapping projections of e.g., fibula or fixation material were included.

#### Callus formation as callus projection size: callus size

**Fig. 3:**
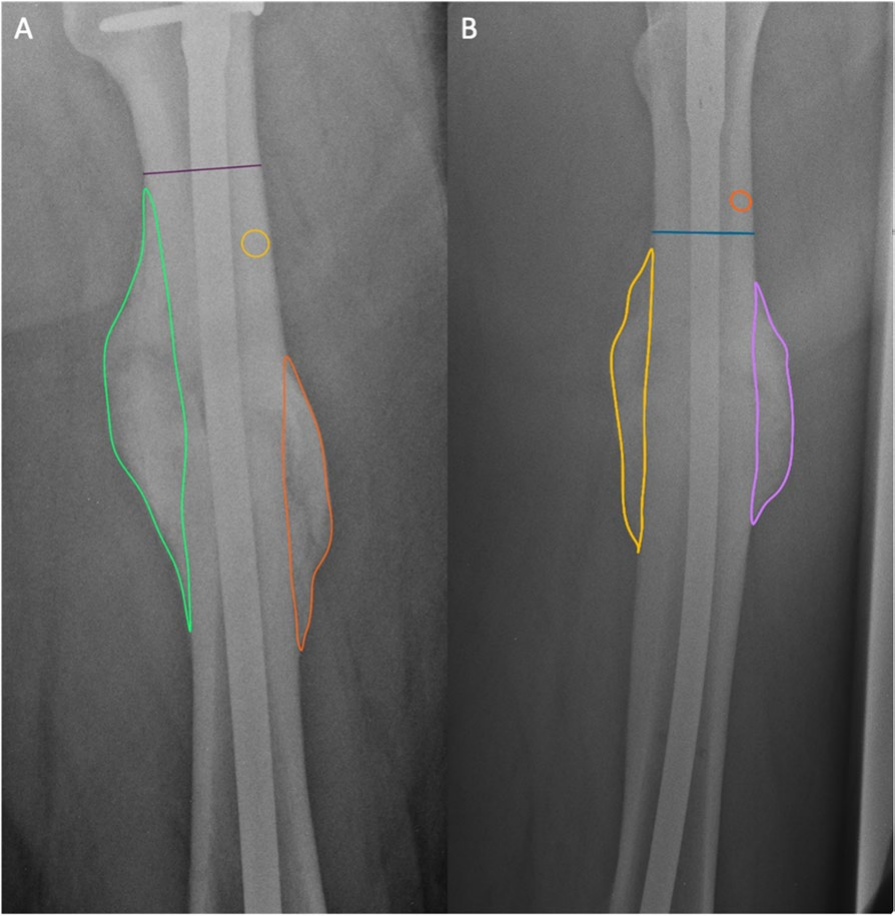
Example of callus size and density evaluation using a femoral shaft fracture stabilized with an intramedullary nail. **A** AP-View with medial callus (green polygon; Area: 14.36 cm^2^; mean density 2369.53 [image intensity]) and lateral callus (orange polygon; Area: 6.85 cm^2^; mean density: 2193.17) as well as shaft width (purple line; 3.39 cm) and cortex density (yellow circle: 2661.9) for normalization. **B** Lateral View with ventral callus (yellow polygon; Area: 9.21 cm^2^; mean density: 1507.36) and dorsal callus (purple polygon; Area: 8.42 cm^2^; mean density: 1763.38) with shaft width (blue line: 3.73 cm) and cortex density (orange circle: 1992.08). For normalization, the callus area was divided by shaft width (normalized callus size = callus area (in cm^2^)/shaft width (in cm)) and callus density by cortex density (normalized density = callus density/cortex density)

Callus sizes were quantitively assessed for each cortical site (medial, lateral, ventral, dorsal) on all available images using the region of interest (ROI) tool “closed polygon” for size measuring (in cm^2^) in a DICOM-viewer “Horos” (The Horos project, V. 3.3.6) (Fig. 3). A similar method has been described before [24, 25]. Bone shaft widths were assessed at the middle shaft and were used for size normalization: normalized callus size = callus area (in cm^2^)/shaft width (in cm), (Fig. 3).

#### Callus quality from image intensity as surrogate for density: callus density

The mean image intensity of each callus ROI was determined. Previous studies have shown a high correlation between mean image intensity (grayscale value) and bone mineral density, suggesting this method is a reliable alternative to DEXA for assessing radiographic density [39]. A reference circle ROI was placed at the lateral/ventral cortex in the proximal third of the bone shaft in a cortex acting as a reference density for unaffected bone (Fig. 3). Normalized density was calculated as the ratio of callus density to cortex density.

#### Quantifying healing progression: mRUST

The modified radiographic union scores for tibia fractures (mRUST) were assessed by rating callus formation and fracture line (callus absent, fracture line visible: 1; callus present, fracture line visible: 2; callus bridging, fracture line visible: 3; callus remodelled, fracture line invisible: 4) at each cortex (frontal and lateral view) (ranged from 4–16) for each timepoint (Fig. 2), [27, 40, 41].

### Matched in-silico analyses of interfragmentary tissue straining

**Fig. 4:**
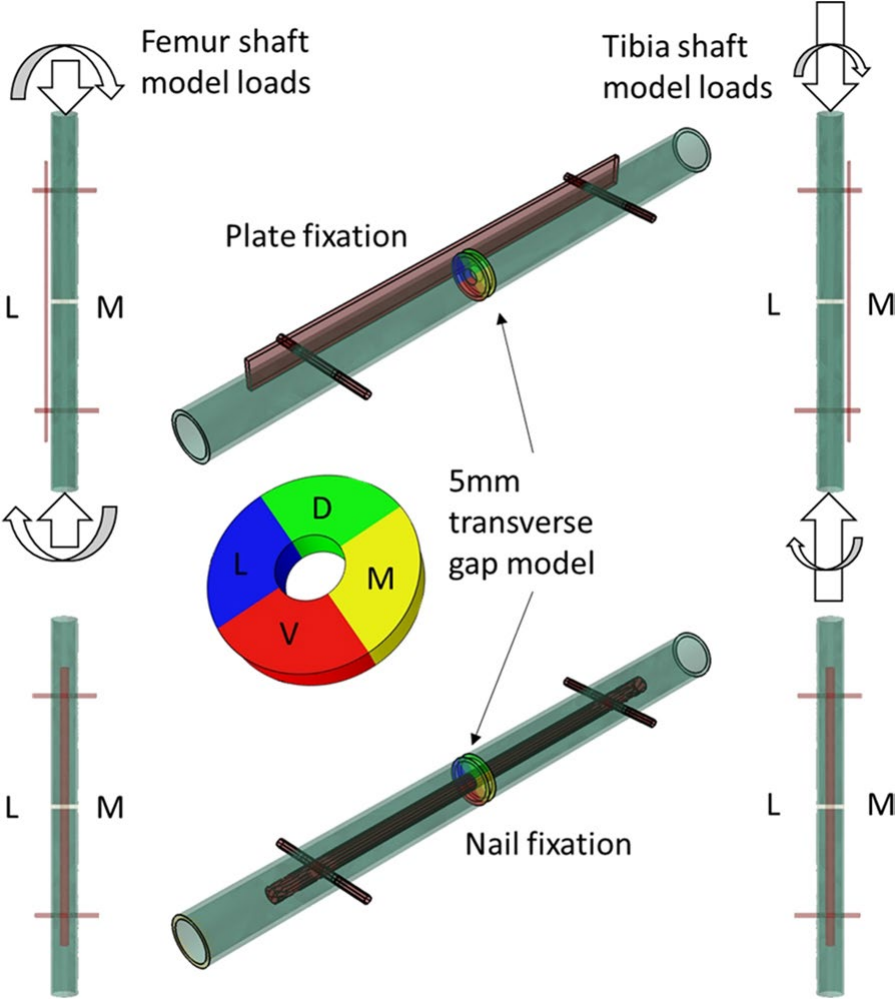
Idealized finite element models. In clinical setting at least two screws per fragment are used, but here with only one screw at each fragment the rotational forces are still controlled, because we also model ideally stiff interactions of the screws to bone and implants. Overall, there is a similar stability compared to the clinical setting, which was kept constant for the same fixation types. Top: Plate fixation, for the femur (left), the plate was positioned laterally (L); for the tibia (right), the plate was placed medially (M). Bottom: Nail fixation was assumed to be symmetrical and no geometrical difference were made for long bone settings at femur or tibia. Each model was loaded accordingly with their respective loads as schematically illustrated on top left with femur loading versus top right with tibial loading (Table 1, [17]). A mid-shaft 5 mm transverse fracture gap filled with soft regenerative tissues was modelled (L: lateral, M: medial, V: ventral, D: dorsal)

**Fig. 5:**
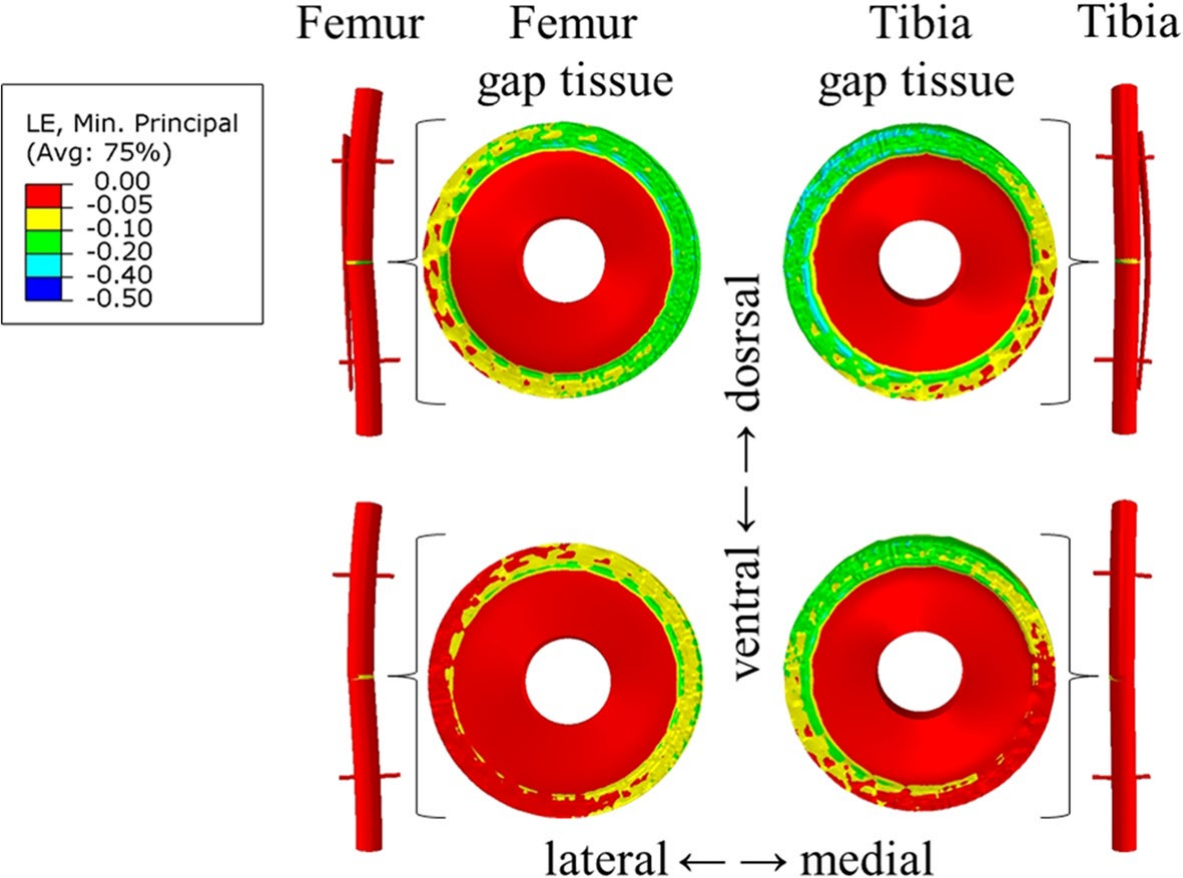
Finite element strain results of different fixation options (top: plating, bottom: nailing; 5 × exaggerated deformation) and different bone loading scenarios (left: femur loading, right: tibia loading; respectively), centrally, the gap tissue minimum principal strain is shown with the dorsal aspect on top, ventral at the bottom, lateral aspect to the left and medial aspect to the right

Separately from the retrospective X-ray assessment, and using finite element analyses (FEA) in 3D, we modelled reproducible mid-shaft fracture fixations with minimal assumptions (Fig. 4), which approximated straining via FE simulation and quantified local deformation patterns for different fracture localizations and different fracture fixations (Fig. 5). To exclude interferences with initial bony support (comminuted fractures versus simple fracture types), we modelled the post-operative setting with a mid-shaft 5 mm gap setting. With this, the analyses focus only on the stability provided by the fixation implants alone and does not overestimate beneficial effects by additional bony support, ideal reduction or muscular stabilization that would help enhancing healing. The mechanical boundary conditions of loading and fracture fixation were matched to meet the specificity of each clinical case based on estimates of bone loading (femoral or tibial loading during walking; [17], Table 2) and using idealized fracture fixation implants. The top part was loaded with the specific loads without any follower load, and the bottom was rigidly fixed and created equal, but opposite reaction forces (Fig. 4, top). The calculation was geometrically non-linear.

**Table 2:** Bone-location matched loading for the femur and tibia according to the derived bone loads for normal walking kinematics from Heyland et al. [17] from a validated musculoskeletal model and load data measured with telemetric knee implants

Loading	Femur shaft loading model		Tibia shaft loading model	
Axial Compression	2 BW	1500 N	4 BW	3000 N
Medio-Lateral Bending	0.1 BW mm	−75 Nm	0.03 BW mm	−22.5 Nm
Postero-Anterior Bending	0.025 BW mm	−18.75 Nm	−0.15 BW mm	112.5 Nm
Torsion	−0.025 BW mm	18.75 Nm	0.03 BW mm	−22.5 Nm

Relative rehabilitation loads in patients are probably smaller due to reduced gait speed and the use of walking aids such as crutches or a walker. To allow comparisons, we referred all analyses to a body weight (BW) of 75 kg across all analyses

For locking plate (300 mm length, 20 mm width, 3 mm thickness) or intramedullary nail (300 mm length, 10 mm diameter) fixation, corresponding elements were generated and placed in the model. In both fixation settings, a proximal and distal bi-cortical locking screw (5 mm diameter, bridge span: 237.5 mm) was modelled and a rigid fixation to the bone (200 mm length per bone fragment, 405 mm total length 2.5 mm cortical thickness) of either the plate or nail was assumed. Screw-bone interface was modelled as fully bonded and the micromotion that may occur at this interface was not modelled; a tied coupling was assumed. A fixed screw-approach may lead to overestimation of the stiffness predictions [42], which we compensate by modelling only one screw-bone interface [18]. Implants material properties were defined with properties of steel material (isotropic, homogeneous with Young’s modulus of 180 GPa), bone material was given Young’s modulus of 17 GPa. As material in the fracture, gap soft tissues were modelled with a modulus of 0.1 GPa, with Poisson ratio of 0.3. In total four different FE models were created (Fig. 4). The models were verified to yield consistent results for different gap tissue model (isotropic or orthotropic) and different gap mesh (hexahedral or tetrahedral) and different mesh sizes.

We evaluated the three principal strain components as well as the compound strain of dilatation (average of principal strain, i.e. measure of the volume change) and distortion (deviatoric strain, i.e. deviatoric strain + volumetric change = total strain) at the four segments medially, laterally, dorsally and ventrally (Fig. 4). Negative strain values were evaluated as positive values in the correlation analysis to simplify interpretation of results: positive coefficients mean a larger strain corresponds to beneficial outcome.

### Statistical analysis

In order to compare standard clinical follow-up time spans, specific time points, i.e. T1-T4, were chosen for evaluation (T1: 90 (± 45 d) days post-surgery, T2: 180 (± 45 d) days post-surgery, T3: 365 (± 45 d) post-surgery, T4: 730 (± 45 d) days post-surgery). The results of the X-ray evaluation are given as descriptive statistics.

For statistical association of retrospective clinical image-based outcome with simulation model output, we performed statistical correlation and regression analyses of callus size, callus density, mRUST and tissue straining across all patients for time points T1 = 90, T2 = 180, and T3 = 365 days post-surgery using IBM SPSS Statistics v29. We evaluated the bi-variate Kendall-Tau-b correlation, and a linear multi-variable regression analysis with inclusion criterion including the patient characteristics of age, body weight, height, BMI, bone localization, and fixation type and the strain components and associated those with callus size, callus density, and the interaction of callus density*size, and mRUST.

## Results

### Patients

From 4,841 patients that were screened, 166 patients qualified to be included into this analysis. Main exclusion criteria were lack of follow-up or insufficient image quality, or intra-articular fractures. The remaining 166 patients were analysed according to their characteristics with more male patients included in both femur and tibial fractures (*n* = 112), mainly high energy trauma causes (*n* = 122), and the majority fixed with nail fixation (*n* = 103; Table 3). We did not consider revision cases or periprosthetic fractures here.

**Table 3:** Patients’ characteristics

	**All patients (*n* = 166)**	**Femur (*n* = 63)**	**Tibia (*n* = 103)**
Sex			
Male	112 M (67.47%)	43 M (68.25%)	69 M (66.99%)
Female	54 F (32.52%)	20 F (31.74%)	34 F (33.01%)
Age	43.4 (SD: 15.75; 18–84)	41.22 (SD: 16.68; 18–84)	44.73 (SD: 15.09; 18–78)
Weight (kg)	81.41 (SD: 15.81; 46–118)	80.03 (SD: 15.85; 46–110)	82.13 (SD:15.87; 52–118)
Height (m)	1.76 (SD: 0.09; 1.53–1.94)	1.74 (SD: 0.09; 1.58–1.88)	1.77 (SD: 0.08; 1.53–1.94)
Side			
Right	87 (51.2%)	34 (53.97%)	53 (51.46%)
Left	79 (47.59%)	29 (46.77%)	50 (48.54%)
Trauma			
High-energy	122 (73.49%)	56 (88.89%)	66 (64.08%)
Low-energy	44 (26.51%)	7 (11.11%)	37 (35.92%)
Fixation			
Nail	103 (62.05%)	44 (69.84%)	59 (57.28%)
Plate	42 (25.3%)	16 (25.4%)	26 (16.5%)
Nail + Plate	18 (10.84%)	1 (1.59%)	17 (16.5%)
Other	3 (1.81%)	2 (3.17%)	1 (0.97%)

A total of 166 patients, whereas 63 patients with femur fractures and 103 patients with tibia extra-articular fractures were included in this analysis. Groups were comparable regarding sex, age and height/weight. Please note that femur fractures were more frequently caused by high energy trauma compared to tibia fractures. For both groups, nail fixation was the most common treatment

### Quantification of callus size and density by X-ray assessment

#### Femoral fracture settings

Fifty-four patients of 63 with femur fractures had imaging at the given specific time points (four patients with only one follow-up < 45 days post-surgery; 5 patients with follow-ups not matching the given timepoints). 38 patients showed callus formations at the medial site of the cortex, 32 laterally, 32 ventrally and 31 dorsally.

##### Callus size

Mean callus sizes including standard deviations in the femur are shown in Table 4 on the left. At T1, highest mean callus size was measured dorsally (0.73 cm^2^/cm), followed by medial callus (0.69 cm^2^/cm), lateral (0.61 cm^2^/cm) and ventral (0.49 cm^2^/cm). Overall, the largest callus size was measured at T2 with mean dorsal callus size of 1.41 cm^2^/cm, medial callus of 1.18 cm^2^/cm, lateral callus of 0.96 cm^2^/cm and ventral callus of 0.73 cm^2^/cm. At T3, callus sizes reduced dorsally (0.93 cm^2^/cm), medially (1.15 cm^2^/cm) and ventrally (0.67 cm^2^/cm), while lateral callus size slightly increased (0.98 cm^2^/cm). Callus size further decreased at T4 with the largest callus medially (0.26 cm^2^/cm), laterally (0.9 cm^2^/cm) and ventrally (0.29 cm^2^/cm), but there were only few patients included at this late follow-up time point. Due to a lack of follow-up at T4, we do not include this time point in the correlation and regression analyses.

**Table 4:** Mean callus size and standard deviation for femur and tibia cases during the four defined time points

Femur callus size	Medial	Lateral	Ventral	Dorsal	Partial eta-squared	Tibia callus size	Medial	Lateral	Ventral	Dorsal	Partial eta-squared
T1	0.69 ± 0.57	0.61 ± 0.42	0.49 ± 0.53	0.73 ± 0.93	0.076	T1	0.31 ± 0.28	0.39 ± 0.27	0.19 ± 0.17	0.3 ± 0.22	0.244
T2	1.18 ± 1.11	0.96 ± 0.62	0.73 ± 0.91	1.41 ± 1.57	0.086	T2	0.45 ± 0.34	0.53 ± 0.35	0.31 ± 0.3	0.47 ± 0.46	0.244
T3	1.15 ± 0.94	0.98 ± 0.5	0.67 ± 0.68	0.93 ± 0.65	0.150	T3	0.46 ± 0.36	0.75 ± 0.49	0.43 ± 0.22	0.44 ± 0.24	0.194
T4	0.26*	0.9 ± 0.34	0.29*	/	N/A	T4	0.26 ± 0.11	0.36 ± 0.28	0.24 ± 0.13	0.29 ± 0.27	N/A

^*^T4 data on callus size for femoral fractures is only given as orientation due to a lack of follow-up data points. Effect size estimates measure of the location at the various time points is given from repeated measures ANOVA as partial eta-squared: eta-squared of 0.02 suggests a small effect, of 0.13 a medium and of 0.26 a large effect

##### Callus density

Mean callus densities including standard deviations in the femur are shown in Table 5 on the left. Mean callus density (relative to cortical bone density) increased steadily from T1 (medial 0.86, lateral 0.88, ventral 0.9, dorsal 0.84) to T2 (medial 0.9, lateral 0.88, ventral 0.96, dorsal 0.87) in all four regions. Callus density exceeded cortex density ventrally (1.02) and dorsally (1.02), but not medially (0.9) and laterally (0.95) at T3. Callus density further increased at T4 (medial 1.14, lateral 1.28, ventral 1.18), but there were only few cases included. Thus, we also excluded T4 for callus density in the correlation and regression analyses.

**Table 5:** Mean callus density and standard deviation for femur and tibia cases during the four defined time points

Femur callus density	Medial	Lateral	Ventral	Dorsal	Partial eta-squared	Tibia callus density	Medial	Lateral	Ventral	Dorsal	Partial eta-squared
T1	0.86 ± 0.16	0.88 ± 0.25	0.9 ± 0.32	0.84 ± 0.2	0.183	T1	0.75 ± 0.28	0.8 ± 0.23	0.98 ± 0.2	0.74 ± 0.2	0.532
T2	0.9 ± 0.22	0.88 ± 0.28	0.96 ± 0.3	0.87 ± 0.13	0.076	T2	0.88 ± 0.37	0.83 ± 0.16	0.97 ± 0.32	0.87 ± 0.28	0.589
T3	0.9 ± 0.21	0.95 ± 0.28	1.02 ± 0.31	1.02 ± 0.25	0.333	T3	0.84 ± 0.23	0.83 ± 0.22	0.82 ± 0.29	0.94 ± 0.25	0.293
T4	1.14*	1.28 ± 0.02	1.18*	/	N/A	T4	0.68 ± 0.06	0.92 ± 0.03	/	/	N/A

^*^T4 data on callus density for femoral fractures is only given as orientation due to a lack of follow-up data points

##### Quantifying healing progression

**Fig. 6:**
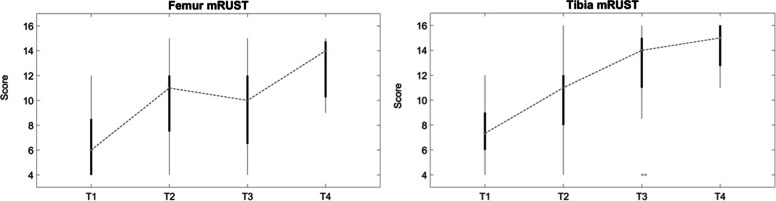
Fracture bridging score outcome of mRUST for femur and tibia cases. The initial healing rate (trendline slope of mRUST increase) is 1.2% higher in tibia fractures compared to femur fracture from T1 to T2. From T1 to T4, the healing rate in tibia fractures is 48.4% higher compared to femur fractures. Numerical values are listed in Supplementary Table S1

The radiological scoring illustrating healing by mRUST increased from T1 (6.72) to T2 (9.62) and decreased slightly at T3 (9.5), due to fewer and potentially biased follow-up at the given time point, while mRUST further increased at T4 (12.67), (Fig. 6, left).

#### Tibial fracture settings

Ninety-four patients of 103 with tibia fracture were identified with imaging at the given time points (five patients with only one follow-up < 45 days post-surgery; 4 patients with follow-ups not matching the given timepoints). 43 calluses were identified medially, 37 laterally, 30 ventrally and 32 dorsally.

##### Callus size

Mean callus sizes including standard deviations in the tibia are shown in Table 5 on the right. Mean maximum relative callus size for all available images was 0.37 (SD 0.1) cm^2^/cm for medial callus, 0.51 (SD 0.18) cm^2^/cm for lateral callus, 0.29 (SD 0.1) cm^2^/cm for ventral and 0.38 (SD 0.09) cm^2^/cm for dorsal callus. For T1 highest mean callus size was measured laterally (0.39 cm^2^/cm), followed by medial callus (0.31 cm^2^/cm), dorsal (0.3 cm^2^/cm) and ventral (0.19 cm^2^/cm). Overall highest callus size was measured at time point T2 with lateral callus size of 0.53 cm2/cm, dorsal callus of 0.47 cm^2^/cm, medial callus of 0.45 cm^2^/cm and ventral callus of 0.31 cm^2^/cm. At T3, callus size kept increasing laterally (0.75 cm^2^/cm), medially (0.46 cm^2^/cm) and ventrally (0.43 cm^2^/cm), while a slight decrease was noted dorsally (0.44 cm^2^/cm). At T4 all sides showed a decrease: medially (0.26 cm^2^/cm), laterally (0.36 cm^2^/cm), ventrally (0.24 cm^2^/cm) and dorsally (0.29 cm^2^/cm). Highest decrease is related to the lateral side with an overall decrease of 48% (Table 4 on the right).

##### Callus density

Mean callus densities including standard deviations in the tibia are shown in Table 5 on the right. Callus density increased steadily from T1 (medial 0.75, lateral 0.8, ventral 0.98) to T2 (medial 0.88, lateral 0.83, dorsal 0.87) for all regions except for the ventral one (slight decrease from 0.98 to 0.97). In T3 all densities decreased with respect to T2 excluding lateral region (keeping the same value), with values of 0.84, 0.83, 0.82 and 0.94 (respectively for medial, lateral, ventral and dorsal side). At the last time point, a lack of follow-up data for ventral and dorsal callus emerged, so only a further decrease of medial (0.68) and increase of lateral one (0.92) could be noted (Table 5 on the right).

##### Quantifying healing progression

The radiological healing mRUST increased constantly over the time points from T1 (7.42), T2 (10.37), T3 (12.96), to T4 (14.22), (Fig. 6, right).

#### Comparison of femur vs. tibia

##### Callus size

Callus sizes in femur were overall higher compared to callus sizes in tibia with mean differences of 0.45 cm^2^/cm medial, 0.36 cm^2^/cm lateral, 0.25 cm^2^/cm ventral and 0.65 cm^2^/cm dorsal across all time points. Highest differences between callus sizes were observed at T2 (2.52 cm^2^/cm), followed by T3 (1.66 cm^2^/cm) and T2 (1.33 cm^2^/cm), while differences at T4 were low (0.3 cm^2^/cm).

##### Callus density

Mean callus density was slightly higher for all regions in femur calluses compared to tibia with a mean difference of 0.16 medially, 0.15 laterally, 0.09 ventrally and 0.06 dorsally across all time points. Mean overall density of the callus in femur and tibia differs by 0.45 at T1, 0.36 at T2, 0.25 at T3 and 0.65 at T4, however not enough data points are available at the last time point T4. Callus density exceeds cortical density (density > 1) in femur fractures at T3 ventrally and dorsally and at T4 medially and laterally, however not in tibia fractures (max. 0.97 ventral at T3). Callus density at femur showed a roughly continuous rise, however for the tibia, only small changes during follow-up were registered, with overall slight increasing from T1 to T2 (except for ventral).

##### Quantifying healing progression

The radiological healing mRUST showed higher values in tibia at all timepoints with differences of 0.5 at T1, 0.75 at T2, 3.46 at T3 and 1.55 at T4. The slope of a linear trendline of T1-T4 as a surrogate of a healing rate (delta mRUST per day) was 0.0093 for femur and 0.0138 for tibia (+ 48%).

### Finite Element Analysis (FEA) strain component correlations with callus density and size

There is an interaction of the relative position in the bone with the fixation type and bone loading: the largest callus appeared dorso-medially in the femur (Table 4), while plates were attached laterally in the femur; largest callus emerged laterally in the tibia (Table 4), while plates were attached medially in the tibia shaft (Table 4, Fig. 5). This effect is similar for the intramedullary nailing where highest loading occurred medially in the femur and dorso-laterally in the tibia. Values of the resultant strain components are defined by the loading and influenced by the relative locations in the femur or tibia and also by the type of fixation chosen (Fig. 5). The overall straining (median of min./max. principal strain −3.77%/−3.80% respectively, median of distortion −0.6%) in the fracture gap appeared to be only slightly smaller with nail fixation compared to plate fixation (Fig. 5). Median strains in tibia gap tissue were higher for the components of maximum principal strain (+ 24.2%) and distortion (+ 10.6%), but slightly lower with minimum principal strain (−2.8%) compared to femoral gap tissue straining.

**Fig. 7:**
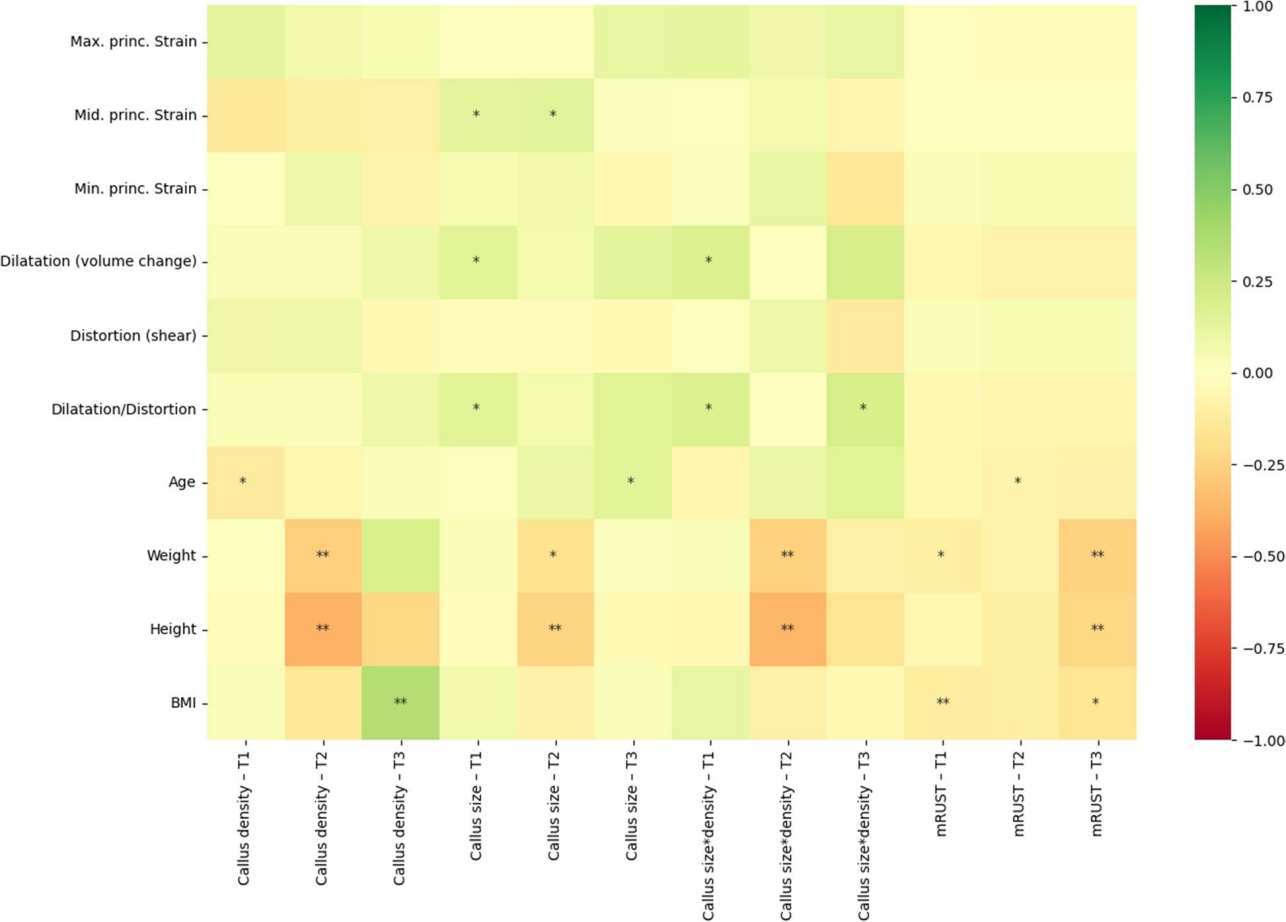
Bi-variate Kendall-Tau-b correlations for the associations of strain components (from finite element model), and patient characteristics age, weight, height, and BMI to callus density, callus size and callus density*size for time points T1, T2, and T3. Significant correlations according to Kendall-Tau-b are marked (*p < 0.05, ** *p* < 0.01)

The strongest bi-variate Kendall-Tau-b correlations were found between callus density at T2 and patient height (*r* = −0.378), between callus size at T2 and patient height (*r* = −0.242), between callus size*density at T2 and patient height (*r* = −0.372), and between mRUST at T3 and patient weight (*r* = −0.257) (Fig. 7). There are only weak, but a few significant correlations of strain components and callus size, and callus size*density (Fig. 7).

Distortion of gap tissues (shear, i.e. strain without volume change) proved to be a negative predictor for ultimate outcome of fracture healing (dilatation/distortion is associated with callus size*density at T1 and T3, bi-variate correlation in Fig. 7), while early callus formation at T1 is associated with larger shear or generally larger strain (multi-variable regression in Table 6). Taken together in a multi-variable linear regression, there was no clear dominant strain component that was significantly associated with callus density, callus size, callus density*size, or mRUST. Patient factors such as age, weight, height or patient BMI showed significant associations with imaging results, which were not robust over all time points (Table 6).

**Table 6:** Multi-variable linear regression analyses for the associations of strain components (from finite element model) and patient characteristics age, weight, and height to callus density, callus size and callus density*size for time points T1, T2, and T3

Linear Regression Model beta coefficient (standardized influence)	Callus density			Callus size			Callus size*density			mRUST		
	T1	T2	T3	T1	T2	T3	T1	T2	T3	T1	T2	T3
R^2^	0.272	0.574	0.508	0.304	0.425	0.282	0.318	0.501	0.324	0.112	0.135	0.490
N (data points)	60	38	27	78	66	47	60	37	27	184	116	104
Max. princ strain	0.106	−2.169	0.000	**−9.741**	−6.297	−1.528	0.475	1.969	0.000	0.000	0.000	0.000
Mid. princ strain	−0.297	−0.625	0.091	**−11.876**	−7.793	−4.071	0.210	0.996	−1.646	0.000	0.000	0.000
Min. princ strain	−0.124	0.000	−0.12	**−13.139**	−7.588	−5.166	−0.037	0.000	−4.304	0.000	0.000	0.000
Dilatation (volume change)	0.000	0.000	0.442	**15.619**	9.868	3.484	0.000	0.000	2.168	0.000	0.000	0.000
Distortion (shear)	−0.053	3.185	0.081	**3.235**	2.514	−1.578	−0.267	−1.814	−2.745	0.000	0.000	0.000
Dilatation/ Distortion	0.000	1.943	0.000	0.000	0.000	0.000	0.000	−2.434	0.000	0.000	0.000	0.000
Age	−0.189	0.091	0.104	0.092	**0.471**	0.104	−0.013	**0.487**	0.100	0.070	−0.023	**−0.230**
Weight	2.196	2.988	3.224	**−4.515**	−3.173	5.183	**−5.779**	−4.245	5.081	0.685	0.572	**−2.294**
Height	−1.186	−1.968	−1.185	**1.903**	0.922	−1.757	**2.348**	1.501	−1.646	−0.530	−0.582	0.699
BMI	−1.470	−2.592	−2.417	**4.064**	2.774	−4.881	**5.238**	3.519	−4.438	−0.640	−0.194	1.717
Bone	0.000	0.000	0.000	0.000	0.000	0.000	0.000	0.000	0.000	0.000	0.000	0.000
Fixation type	0.056	**−0.992**	0.314	**0.314**	0.373	0.035	0.227	−0.521	0.008	**0.332**	**3.643**	**3.779**

Significant correlations are marked **bold (*p* < 0.05)**

In the multi-variable regression, minimum principal strain was also strongly colinear with other strain components (Supplement Figure S1), so the additional predictive value of the minimum principal strain data was low (Table 6). Callus size could not be well predicted, i.e. overall low *R*^2^ values, and strain components were not very influential and showed few significant correlations. The associations of callus size*density were similar, but with higher *R*^2^ values. Healing with mRUST could only be predicted (including all parameters, Table 6 shows only the best regression model after excluding weak predictive parameters) with moderate R^2^ for T3 with a significant negative predictive capacity of age (beta = −0.248, i.e. a relatively weak effect) and weight (beta = −2.298, i.e. a relatively weak effect) compared to much stronger standardized effects of the strain with beta = 11.647, 12.071, −9.038, and −19.143 for strain components of max. principal strain, mid. principal strain, distortion, and dilatation/distortion respectively.

## Discussion

In this study, callus formation and quality during X-ray follow-up of extra-articular femoral and tibial fractures were assessed and correlated with modelled mechanical strain components. Therefore, lower-limb fractures stabilized with plate or intramedullary nail were analysed in their specific mechanical treatment and localization setting using finite element analyses and correlated with the imaging outcome. Among other factors, mechanical conditions are known to influence the callus formation, but no direct robust links between the mechanical environment and patient outcome have been shown so far [43]. Multiple studies show the formation of asymmetrical and inconsistent callus [24, 25, 44]. Specifically, long bone fractures of the lower limb experience significant forces and moments [17]. These loads result in local mechanical constraints to the healing, represented by interfragmentary straining of the fracture site, eventually leading to interfragmentary movements of up to a few millimeters [18, 19, 21, 22, 26, 45–48]. We hypothesized that the local strain in a fracture gap is an invariant parameter that affects callus formation and quality as documented by X-ray follow-up of such fracture healing processes. The precise strain values or components such as compressive or principal strain and shear strain acting at a fracture and their thresholds differentiating between beneficial mechanical conditions for healing have been discussed controversially [6, 31, 32, 34–37].

In this retrospective analysis of 166 clinical fracture cases, we could identify differences in the strain components according to the fractured bone (femur or tibia), and within a callus (medially, laterally, dorsally, or ventrally) and due to the chosen fixation (Fig. 5), in line with the asymmetric callus formation described by [24, 25]. In general, mechanical strain values appeared to be higher in tibia fractures compared to femur fractures and resulted in smaller and less dense callus compared to femur fracture callus (Fig. 5, Tables 4 and 5). While in femur fractures, the largest callus can be found dorso-medially (with plates usually located laterally), in tibia fractures, the largest callus appeared laterally (with plates usually located medially). Strain components derived from load- and fixation-matched finite element analyses correlated with callus size (*p* = 0.019–0.036). Individual bi-variate predictive values of individual strain components appear to be weak. This corresponds to published data where univariate linear regression of callus formation was not significant, but multi-variable regression showed significant strong coefficients for shear (detrimental) and compressive (beneficial) interfragmentary movement for callus sizes [22]. However, this earlier investigation relied on a smaller patient cohort (*n* = 66 patients) and restricted itself to distal femur plate fixation. However, they accounted for different fixation stiffness.

Individual, more patient-specific strain values for given fracture settings were not achievable in the present setting and require more in-depth details of the specific cases and may be subject to future prospective studies. For individual patient- and fracture-specific strain analyses, gap dimensions, individual tissue properties, specific fixation stiffness, and loading characteristics would need to be accounted for. However, local strain estimates based on FEA and patient records allow to have an insight of local callus development. While shear strain (distortion) was consistently detrimental and associated with smaller callus, maximum and middle principal strains showed rather beneficial effects, which is probably related to the longitudinal motion that has been described as beneficial by Elkins et al. [22]. This finding is strongly supported by the scoping review from Rechter et al. [38], which concluded that while shear motion is largely regarded to possess an inhibitory impact on callus formation, controlled small-to-moderate axial interfragmentary motion (a driver of principal strain) is associated with superior stiffness and callus proliferation. Our results, drawn from a large clinical cohort, provide direct clinical validation for this distinction. This confirms the use of the ratio of dilatational/distortional strain in existing mechanobiological models [6, 31, 32, 34–37].

However, more details on ranges of strain components in principal strain and shear for ideal fracture fixations remain to be determined in prospective clinical trials with details on the exact fracture location, gap dimensions and eventual material property [49]. While we did not explicitly model callus tissue maturation and size increase in the study presented here, the early phase of bone healing is characterized by rather constant mechanical conditions, specifically periosteally. We idealized the bone shapes to same-size circular cross sections, and in case of parts of the tibia with a rather triangular outer cross section, strain patterns might deviate locally, but for correlation analysis, we used the mean values of the strain distribution over a specified area (medial, lateral, posterior, anterior) and during callus tissue maturation, the newly formed callus tends to form circular cross sections. Our idealized model is also rather a model of diaphyseal fractures, while most indications for plating are rather metaphyseal fractures. Analyzing the mechanical conditions using a FEA model, multiple variables are considered: Mechanical simulations were made in two fixation models for each bone localization (femur or tibia), which limits the generalizability. There are several further variables such as numbers of screws or their position, which influence the mechanical environment. Furthermore, the increasing callus size and its compensating effect were not considered in the model. As we investigated different bones and different locations within the bones, bone shaft widths and bone densities differ between patients. We wanted to control for any sizing effects that could influence callus size or density. We expect a generally larger callus (volume) with a larger bone circumference and a denser callus with a denser overall bone shaft, and thus normalized our measurements by bone width and bone shaft density. Also, the used implants and loads are idealized and their effects might differ in situ. However, the model still showed a clear trend of strains and model parameters were chosen according to the literature and current research [17]. This idealized approach is a clear limitation. It contrasts with patient-specific, longitudinal finite element models, such as the work by Vijayakumar et al. [50], which meticulously tracked the decreasing load share of an external fixator from 20 to 2% as callus stiffness increased in a single patient. Such patient-specific, time-dependent modeling was outside the scope of this large-scale retrospective study, which aimed to identify generalizable correlations across a broad, heterogeneous cohort rather than produce predictive models for individual patients. The X-ray assessment was performed by only one reader and even small angulation of the X-ray image led to different callus size. There is a lack of availability of follow-up data, especially in older patients which leads to a relatively young study population and potential selection bias. Further prospective studies with standardized, ideally more detailed imaging at given follow-up time points are needed with even closer documentation of the identified relevant mechanical parameters [51].

The current analyses illustrate that callus size or callus density alone are not sufficiently reliable predictors of healing, as there is a discrepancy between healing rates (mRUST increase) in tibia and femur fractures and the callus size and density development. In our analysis, the healing process showed different trends: While tibia fracture calluses tended to increase constantly over time, femoral fractures appeared to develop slower and reaching lower values of mRUST at the last follow-up. Higher healing rates were shown for the tibia compared to the femur. For the tibia, higher max. principal strain and distortion were found compared to the femur. The higher distortion in the tibia (around 10%) might explain the smaller and less dense callus in the tibia. Surprisingly, the mRUST increase over time was higher in the tibia compared to the one in the femur. We assume that the high compression loading of the tibia compared to the femur [17] results in overall dominating (compressive) strains and minimized shear in this bone. The initial healing rate appears similar between femoral and tibial fractures (Fig. 6), expressed by pronounced callus formation between T1 and T2 (Table 4), but this seemed not to result in better healing outcome later on in the femur fracture cases. This is consistent with earlier pre-clinical work [15]. However, there were relatively low patient numbers in the specific groups, with different follow-up numbers in the different groups. In our investigation, there was no differentiation between diaphyseal vs. metaphyseal fractures or different fracture types.

In the future, patient-specific finite-element modelling [52] could allow a better match of the postoperative radiographs with the model results, or even reach predictive power to allow estimates of the later healing development, consolidation or implant selections [5, 53–56] eventually resulting in pre-clinical or clinical in silico trials [51, 57]. Our approach could be employed to unravel the mechanical conditions of delayed or non-union cases to identify the underlying mechanical causes of such delays in healing. Such analyses could not only explain failures of healing better but also allow to assess a planned method of fixation in revisions if needed. Furthermore, these predictive models could be validated using emerging in-vivo measurement systems, such as fluoroscopy or dynamic load-share ratio systems [30, 58–61]. This would help bridge the gap between simulated mechanics and objective clinical decisions, such as determining the optimal timing for fixator removal. Patient-specific analyses of fracture conditions and treatments could be optimized to the need of the intra-operative setting of a selected fracture and its fixation and thus surgical decision making could be guided.

In this analysis, we showed how callus appearances are correlated to mechanical strain in extra-articular lower limb fractures. Callus development gives valuable information about the mechanical strain in the fracture gap, which explains their clinical usefulness, and this could lead to more adapted fracture fixation for more consistent and robust fracture healing.

## Supplementary Information

Supplementary Material 1: Figure S1. Bi-variate Kendall-Tau-b correlations for all associations and co-correlations of strain components (from finite element model), and patient characteristics age, weight, height, and BMI to callus density, callus size and callus density*size for time points T1, T2, and T3, as well the maximum values (over all time points, including T4). Significant correlations according to Kendall-Tau-b are marked bold (*p < 0.05, ***p < 0.01), number of data points (patients and localizations anterior, posterior, medial, lateral) is given as N in Table S2.

Supplementary Material 2: Table S1. mRUST scores for femur and tibia cases during the four defined time-points T1-T4 (T1: 90 (± 45 d) days post-surgery, T2: 180 (± 45 d) days post-surgery, T3: 365 (± 45 d) post-surgery, T4: 730 (± 45 d) days post-surgery)

Supplementary Material 3: Table S2. Bi-variate Kendall-Tau-b correlations for the associations of strain components (from finite element model), and patient characteristics age, weight, height, and BMI to callus density, callus size and callus density*size for time points T1, T2, and T3. Significant correlations according to Kendall-Tau-b are marked bold (*p* < 0.05), number of data points (patients and localizations anterior, posterior, medial, lateral) is given as N.

## Data Availability

The datasets generated and analysed during the current study are not publicly available due to data protection and privacy regulations but are available from the corresponding author on reasonable request and with appropriate institutional approvals.

## Abbreviations

ANOVA: Analysis of Variance
BMI: Body Mass Index
BW: Body Weight
DEXA: Dual-Energy X-ray Absorptiometry
DICOM: Digital Imaging and Communications in Medicine
FEA: Finite Element Analysis
mRUST: Modified Radiographic Union Score for Tibia fractures
ROI: Region of Interest
SD: Standard Deviation
3D: Three-Dimensional
