# Supplementary material for: Callus formation during healing is guided by local strain: a retrospective clinical observation
Source: BMC Musculoskelet Disord. 2026 Jun 26;27:556. doi: 10.1186/s12891-026-10118-2 (PMC13321769; doi:10.1186/s12891-026-10118-2)
Supplement: Supplementary file 1 — Supplementary Material 1: Figure S1. Bi-variate Kendall-Tau-b correlations for all associations and co-correlations of strain components (from finite element model), and patient characteristics age, weight, height, and BMI to callus density, callus size and callus density*size for time points T1, T2, and T3, as well the maximum values (over all time points, including T4). Significant correlations according to Kendall-Tau-b are marked bold (*p < 0.05, ***p < 0.01), number of data points (patients and localizations anterior, posterior, medial, lateral) is given as N in Table S2. [file 12891_2026_10118_MOESM1_ESM.docx]

**Supplement**


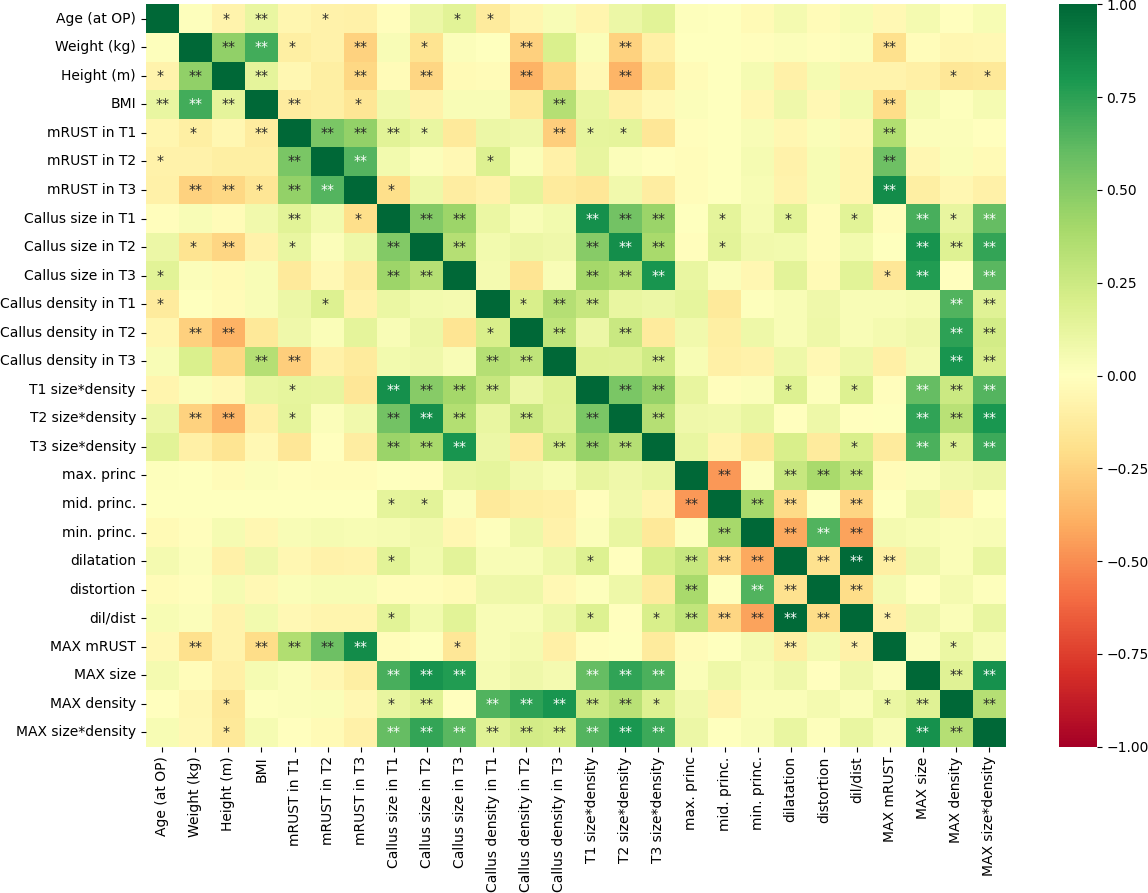


*Figure S1: Bi-variate Kendall-Tau-b correlations for all associations and co-correlations of strain components (from finite element model), and patient characteristics age, weight, height, and BMI to callus density, callus size and callus density*size for time points T1, T2, and T3, as well the maximum values (over all time points, including T4). Significant correlations according to Kendall-Tau-b are marked* ***bold (*p<0.05, ***p<0.01)****, number of data points (patients and localizations anterior, posterior, medial, lateral) is given as N in Table S2****.***
