# Supplementary material for: Callus formation during healing is guided by local strain: a retrospective clinical observation
Source: BMC Musculoskelet Disord. 2026 Jun 26;27:556. doi: 10.1186/s12891-026-10118-2 (PMC13321769; doi:10.1186/s12891-026-10118-2)
Supplement: Supplementary file 2 — Supplementary Material 2: Table S1. mRUST scores for femur and tibia cases during the four defined time-points T1-T4 (T1: 90 (± 45 d) days post-surgery, T2: 180 (± 45 d) days post-surgery, T3: 365 (± 45 d) post-surgery, T4: 730 (± 45 d) days post-surgery) [file 12891_2026_10118_MOESM2_ESM.docx]

**Supplement**

*Table S1: mRUST scores* *for femur and tibia cases during the four defined time-points T1-T4 (T1: 90 (± 45d) days post-surgery, T2: 180 (± 45d) days post-surgery, T3: 365 (± 45d) post-surgery, T4: 730 (± 45d) days post-surgery).*

|  | **mRUST** | |
| --- | --- | --- |
| **Time-points** | **Femur** | **Tibia** |
| T1 | 6.72±2.53 | 7.42±2.2 |
| T2 | 9.62±3.5 | 10.37±2.88 |
| T3 | 9.5±3.49 | 12.96±2.97 |
| T4 | 12.67±3.21 | 14.22±1.86 |
