# Supplementary material for: Callus formation during healing is guided by local strain: a retrospective clinical observation
Source: BMC Musculoskelet Disord. 2026 Jun 26;27:556. doi: 10.1186/s12891-026-10118-2 (PMC13321769; doi:10.1186/s12891-026-10118-2)
Supplement: Supplementary file 3 — Supplementary Material 3: Table S2. Bi-variate Kendall-Tau-b correlations for the associations of strain components (from finite element model), and patient characteristics age, weight, height, and BMI to callus density, callus size and callus density*size for time points T1, T2, and T3. Significant correlations according to Kendall-Tau-b are marked bold (p < 0.05), number of data points (patients and localizations anterior, posterior, medial, lateral) is given as N. [file 12891_2026_10118_MOESM3_ESM.docx]

**Supplement**

*Table S2: Bi-variate Kendall-Tau-b correlations for the associations of strain components (from finite element model), and patient characteristics age, weight, height, and BMI to callus density, callus size and callus density*size for time points T1, T2, and T3. Significant correlations according to Kendall-Tau-b are marked* ***bold (p<0.05)****, number of data points (patients and localizations anterior, posterior, medial, lateral) is given as N****.***

| Kendall-Tau-b  correlation | Callus density | | | Callus size | | | Callus size*density | | | mRUST | | |
| --- | --- | --- | --- | --- | --- | --- | --- | --- | --- | --- | --- | --- |
|  | T1 | T2 | T3 | T1 | T2 | T3 | T1 | T2 | T3 | T1 | T2 | T3 |
| **Max. princ.**  **strain** | 0.130 | 0.075 | 0.046 | 0.001 | -0.012 | 0.114 | 0.123 | 0.080 | 0.112 | -0.015 | -0.022 | -0.022 |
| p-value | 0.094 | 0.366 | 0.657 | 0.990 | 0.855 | 0.155 | 0.113 | 0.345 | 0.277 | 0.710 | 0.647 | 0.700 |
| N | 95 | 86 | 56 | 126 | 136 | 86 | 95 | 84 | 56 | 83 | 60 | 46 |
| **Mid. princ.**  **strain** | -0.139 | -0.106 | -0.098 | **0.137** | **0.148** | 0.022 | -0.012 | 0.070 | -0.063 | 0.000 | 0.000 | 0.000 |
| p-value | 0.073 | 0.201 | 0.342 | **0.037** | **0.019** | 0.788 | 0.876 | 0.402 | 0.540 | 1.000 | 1.000 | 1.000 |
| N | 95 | 86 | 56 | **126** | **136** | 86 | 95 | 84 | 56 | 83 | 60 | 46 |
| **Min. princ.**  **strain** | 0.010 | 0.086 | -0.079 | 0.050 | 0.078 | -0.052 | 0.018 | 0.111 | -0.146 | 0.032 | 0.047 | 0.046 |
| p-value | 0.900 | 0.300 | 0.443 | 0.452 | 0.230 | 0.525 | 0.815 | 0.190 | 0.158 | 0.448 | 0.348 | 0.429 |
| N | 95 | 86 | 56 | 126 | 136 | 86 | 95 | 84 | 56 | 83 | 60 | 46 |
| **Dilatation**  **(volume change)** | 0.037 | 0.032 | 0.089 | **0.151** | 0.068 | 0.147 | **0.172** | -0.003 | 0.201 | -0.054 | -0.079 | -0.077 |
| p-value | 0.633 | 0.702 | 0.387 | **0.021** | 0.281 | 0.067 | **0.027** | 0.974 | 0.051 | 0.192 | 0.108 | 0.176 |
| N | 95 | 86 | 56 | **126** | 136 | 86 | **95** | 84 | 56 | 83 | 60 | 46 |
| **Distortion**  **(shear)** | 0.084 | 0.086 | -0.043 | -0.020 | -0.019 | -0.032 | 0.009 | 0.093 | -0.128 | 0.030 | 0.045 | 0.044 |
| p-value | 0.281 | 0.299 | 0.679 | 0.755 | 0.762 | 0.689 | 0.903 | 0.270 | 0.214 | 0.458 | 0.360 | 0.441 |
| N | 95 | 86 | 56 | 126 | 136 | 86 | 95 | 84 | 56 | 83 | 60 | 46 |
| **Dilatation/**  **Distortion** | 0.038 | 0.031 | 0.090 | **0.152** | 0.068 | 0.150 | **0.173** | -0.003 | **0.203** | -0.046 | -0.067 | -0.066 |
| p-value | 0.621 | 0.705 | 0.382 | **0.021** | 0.281 | 0.062 | **0.026** | 0.974 | **0.048** | 0.265 | 0.170 | 0.248 |
| N | 95 | 86 | 56 | **126** | 136 | 86 | **95** | 84 | **56** | 83 | 60 | 46 |
| **Age** | **-0.128** | -0.058 | 0.032 | -0.011 | 0.095 | **0.151** | -0.067 | 0.100 | 0.149 | -0.055 | **-0.081** | -0.091 |
| p-value | **0.020** | 0.327 | 0.679 | 0.821 | 0.058 | **0.021** | 0.223 | 0.096 | 0.052 | 0.086 | **0.037** | 0.056 |
| N | **156** | 132 | 83 | 189 | 184 | **113** | 156 | 130 | 82 | 118 | **83** | 57 |
| **Weight** | 0.002 | **-0.261** | 0.192 | 0.037 | **-0.179** | 0.017 | 0.031 | **-0.256** | -0.095 | **-0.105** | -0.083 | **-0.257** |
| p-value | 0.979 | **0.005** | 0.113 | 0.571 | **0.016** | 0.855 | 0.661 | **0.006** | 0.439 | **0.019** | 0.155 | **0.000** |
| N | 94 | **58** | 35 | 114 | **88** | 55 | 94 | **57** | 34 | **62** | 39 | **30** |
| **Height** | -0.030 | **-0.378** | -0.227 | -0.026 | **-0.242** | -0.034 | -0.044 | **-0.372** | -0.169 | -0.054 | -0.107 | **-0.228** |
| p-value | 0.672 | **0.000** | 0.064 | 0.689 | **0.001** | 0.725 | 0.544 | **0.000** | 0.177 | 0.231 | 0.071 | **0.001** |
| N | 94 | **58** | 35 | 114 | **88** | 55 | 94 | **57** | 34 | 62 | 39 | **30** |
| **BMI** | 0.036 | -0.143 | **0.336** | 0.076 | -0.086 | 0.033 | 0.114 | -0.094 | -0.043 | **-0.119** | -0.103 | **-0.162** |
| p-value | 0.606 | 0.115 | **0.005** | 0.238 | 0.243 | 0.727 | 0.106 | 0.305 | 0.721 | **0.007** | 0.073 | **0.016** |
| N | 94 | 58 | **35** | 114 | 88 | 55 | 94 | 57 | 34 | **62** | 39 | **30** |
